# WAR as slogan, HAT as strategy: metaphorical construction of poverty alleviation in Chinese political discourse

**DOI:** 10.3389/fpsyg.2026.1821736

**Published:** 2026-09-24

**Authors:** Feifei Fu, Long Dai

**Affiliations:** 1 Zhejiang Business College, Hangzhou, China; 2 Zhejiang Gongshang University Hangzhou College of Commerce, Hangzhou, China

**Keywords:** Chinese political discourse, HAT metaphor, political metaphor, poverty alleviation discourse, WAR metaphor

## Abstract

Political metaphors play a crucial role in shaping public perception and legitimizing policy, yet their use in China’s poverty alleviation discourse remains underexplored. This study investigates the major conceptual metaphors in Chinese governmental discourse on poverty alleviation, drawing on a 33,104-word corpus compiled from official reports over the past decade. Following the Metaphor Identification Procedure VU University Amsterdam (MIPVU) procedure for metaphor identification, the study identifies four dominant source domains: WAR, PATH, COMPETITION, and HAT. Guided by Charteris-Black’s Critical Metaphor Analysis, the study examines how and why these metaphors are strategically employed in the Chinese political context. The analysis reveals that metaphors serve not only to facilitate policy communication and mobilize public support, but also to project a positive national image and position China as a model in global poverty reduction. This study contributes to political metaphor research by identifying the HAT metaphor as a culturally specific framing device in Chinese poverty alleviation discourse, offering both theoretical and practical insights for cross-cultural metaphor studies and policy communication.

## 1 Introduction

China’s poverty alleviation campaign was a defining national strategy launched on a massive scale under the leadership of the Communist Party of China (CPC). Its primary goal was to eradicate extreme poverty nationwide. Officially declared a “complete victory” in 2021 (State Council Information Office of the People’s Republic of China, 2021), the campaign lifted nearly 100 million rural residents out of absolute poverty. The campaign mobilized vast state resources and millions of government officials, who were stationed in impoverished counties to oversee the implementation of policies (Cai and Tang, 2023; Ruan et al., 2024). It is regarded by the Chinese government as a historic achievement and a milestone in its development path (State Council Information Office of the People’s Republic of China, 2021), making a significant contribution to the global reduction of extreme poverty, as claimed by the United Nations (2019).

The campaign generated a substantial body of political discourse, including policy documents, leaders’ speeches, and state media coverage. As Chilton (2025) argues, politics is inseparable from language. There cannot be any politics without language. This fundamental insight underscores the importance of analyzing political discourse, including the ways in which language frames policy issues and shapes public understanding. This premise makes political discourse a crucial site for investigating how specific linguistic devices, such as metaphor, shape public understanding and legitimize state action. Examining this discourse not only illuminates the linguistic mechanisms of policy communication in China but also contributes to broader debates on how political language functions in non-Western contexts.

Metaphor is a fundamental cognitive mechanism through which abstract concepts are made comprehensible (Lakoff and Johnson, 1980). The pervasive use of metaphor in political discourse has drawn sustained scholarly interest, not merely because it adds rhetorical flourish, but because it fundamentally shapes reasoning, legitimizes political decisions, and conditions public perceptions of governance (Charteris-Black, 2004; Musolff, 2016). Political metaphors have attracted extensive scholarly attention across diverse cultural and political contexts. In Western settings, researchers have examined their rhetorical and ideological functions in political communication (Charteris-Black, 2006; Musolff, 2006; Flusberg et al., 2018). In contrast, a growing body of corpus-based research has also explored metaphorical patterns in Chinese political discourse (Lu and Ahrens, 2008; Zeng et al., 2021; Zeng and Ahren, 2023).

Previous studies have examined poverty discourse in China, focusing both on how language reflects social realities and on how poverty is understood and addressed in the Chinese context (Tang and Mu, 2024; Chen, 2026). Given the prominence of poverty discourse on China’s political agenda, one might expect extensive scholarly attention to its metaphorical underpinnings. Yet, despite the analytical potential of China’s poverty alleviation campaign as a site for examining political metaphor use, the strategic deployment of metaphors in this discourse remains largely unexplored. Examining conceptual metaphors for poverty in political discourse offers a productive entry point for understanding how social issues are discursively constructed and conceptualized across different cultural contexts.

This study addresses the gap by examining the deployment and functions of major conceptual metaphors in Chinese political discourse of the poverty alleviation campaign. Drawing on Critical Metaphor Analysis (Charteris-Black, 2004), this study examines the “what,” “how,” and “why” questions concerning the metaphorical framing of poverty in China. Specifically, this study proposes two research questions:

*RQ1:* What source domains dominated the metaphorical framing of poverty alleviation in Chinese political discourse?

*RQ2:* How and why were these dominant metaphors employed in Chinese political discourse?

## 2 Literature review

This study investigates the major metaphors in Chinese political discourse on poverty alleviation. To situate the analysis, we revisit the theory of conceptual metaphor, Critical Metaphor Analysis, previous research on political metaphors, and studies on the metaphorical framing of poverty.

### 2.1 Conceptual metaphor theory and critical metaphor analysis

In our daily practice, the majority of concepts are partially understood in terms of other concepts. Although the metaphor is one of the most important tools for conceptualizing what is unfamiliar or unknown. Conceptual metaphor theory, proposed by Lakoff and Johnson (1980), is the earliest-formed and fundamental theoretical framework to analyze metaphors in cognitive approaches.

Lakoff and Johnson (1980) define metaphor as a device that allows us to understand one domain of experience in terms of another, and as a tool that enables people to use what they know about their direct physical and social experiences to understand more abstract things. We always say “Life is a journey,” but only a few of us realize that the abstract and philosophical concept of “life” relies on a more concrete concept of “journey” in this discourse. Conceptual metaphor theory views metaphors as a way of conceptualizing life as a journey, not just rhetorical or poetic expressions. In this conceptual metaphor, the mapping is from the source domain of a JOURNEY to the target domain of LIFE; within the mapping processes, birth is the start of a journey, death is the end of a journey, and difficulties in life are impediments in a journey. This conceptual metaphor is a way of reasoning about life using the inferential structure of the concept of a journey.

Arguing that metaphor analysis should be a central component of critical discourse analysis, Charteris-Black (2004) proposed the concept of Critical Metaphor Analysis (CMA) in large corpora, which integrates linguistic analysis with cognitive understanding and social insight. Based on Fairclough (1995) three stages of identification, interpretation, and explanation of Critical Discourse Analysis (CDA), Charteris-Black claimed that critical analysis of metaphor could be conducted following the three steps: metaphor identification, metaphor interpretation, and metaphor explanation. In Charteris-Black’s description, we should first aim to identify candidate metaphors by determining whether there is a semantic tension between a literal source domain and a metaphorical target domain. In the second step of metaphor interpretation, we identify conceptual metaphors and conceptual keys, i.e., source domains, target domains, domain mappings, and social relations constructed through them. Third, we identify metaphors’ social roles in persuasion and how they are interrelated and become coherent concerning the situation in which they occur.

Charteris-Black claimed CMA supplements the cognitive theory of metaphor because earlier cognitive semantics studies only focused on “how metaphors are understood” rather than “why they are chosen.” CMA takes into account ideologies underlying metaphors, which may provide a more convincing claim of the purpose of using particular metaphors within specific discourse contexts. Studies in this vein include Charteris-Black (2004, 2006, 2019), Goatly (2007), Musolff (2006, 2012, 2016), and many others.

### 2.2 Metaphor in political discourse

Metaphors are widely acknowledged as a strategic tool for politicians to frame critical issues, with cognitive linguists further identifying their significant ideological functions within political discourse (Ferrari, 2007). Lakoff (1996) claims that in American society, distinct political ideologies are fundamentally constructed upon different metaphorical models.

Employing the analytical framework of CMA, Charteris-Black (2006) analyzes UK political discourse about immigration, arguing that right-wing politicians deploy “natural disaster” and “container” metaphors to advocate for border security and amplify public fear. Goatly (2007) applies CMA to uncover hidden ideologies within the metaphorical patterns of economic and political texts. Musolff’s (2016) research on British political discourse similarly highlights the prevalence of specific metaphorical “scenarios,” such as WAR, FAMILY, BODY, and PERSON, in conceptualizing politics and political entities. Musolff (2006, 2016) analyzed metaphor scenarios in political discourse as European countries, the EU, and mainstream media expressed political attitudes. For instance, the “LOVE-MARRIAGE-FAMILY” scenario was used to describe the relationship between France and Germany. Charteris-Black (2019) identifies CONTAINMENT and GAMES as two dominant, “weaponized” metaphor types in Brexit rhetoric, which influenced both Leave and Remain supporters.

The use of metaphor in political discourse has attracted extensive scholarly attention, yet it remains a subject of considerable debate. On one side of this debate, scholars have drawn attention to the potentially distorting effects of political metaphors. Lakoff (1991) famously cautions that “metaphor can kill,” arguing that WAR metaphors can obscure realities by allowing politicians to glorify war motivations while masking their invariably harmful consequences. On the other hand, a growing body of scholarship has highlighted the constructive functions of political metaphors. Thompson (2018) defends political metaphors as vehicles for fostering group identity and enhancing public participation. Charteris-Black (2011, 2018) further identifies persuasive functions of political metaphor, ranging from rhetorical and heuristic to ideological and mythic. Similarly, Musolff (2016) observes that metaphors carry pragmatic “added values” that shape evaluation, elicit emotion, and reassure the public by reframing threats in familiar terms. This ongoing debate thus calls for further studies that examine the actual functions and effects of political metaphors in concrete discursive contexts.

Meanwhile, scholars have argued that both metaphor use and its interpretation are context-based (Kövecses, 2015; Flusberg et al., 2018; Fu, 2024). Fu (2024) found through empirical research that Chinese participants perceived the WAR metaphor in the Chinese context differently from their American and British counterparts, who largely rejected it (Bates, 2020; Charteris-Black, 2021). Accordingly, the use of political metaphors may differ between Chinese and Western contexts, even in seemingly similar settings. This calls for a systematic examination of metaphorical framing in Chinese political discourse, where contextual factors may shape both the production and reception of metaphors in ways that differ from Western settings.

### 2.3 Metaphorical investigations on social poverty

In the 1960s, the U. S. government, led by President Lyndon B. Johnson, made a series of landmark decisions to address domestic poverty, the centerpiece of which was the launch of the “War on Poverty” campaign. Flusberg et al. (2018) have noted the WAR metaphor in such discourse, observing that “[d]eclaring a War on Poverty directed the public’s attention to the issue and paved the way for important anti-poverty policies and legislation” (p. 7).

Previous studies have taken social discourse on poverty as a key site for investigating how metaphorical expressions are used and understood in the Western context. Among them, Dodge (2016) and David and Matlock (2018) have conducted corpus-based analyses to examine metaphorical expressions related to poverty. Dodge (2016) adopts a combined methodological approach that integrates Conceptual Metaphor Theory, metaphor constructions, and computational tools. These methods are applied within a large-scale corpus-based framework to examine POVERTY metaphors. His findings suggest that when individual metaphors and frames are viewed as components of larger conceptual networks, it becomes possible to capture both local details and broader patterns of metaphor use. David and Matlock (2018) employ Natural Language Processing (NLP) techniques to analyze POVERTY metaphors in large corpora. Their findings further demonstrate that computational methods can reveal shared metaphorical patterns across languages while also highlighting cross-cultural variation.

Yet, despite the growing body of studies on POVERTY metaphors in Western contexts, culturally grounded investigations into Chinese poverty alleviation metaphors remain scarce. This gap is worth addressing, as poverty alleviation has been a long-standing governmental endeavor in China for more than four decades (Liu et al., 2020) and has given rise to an increasingly extensive body of domestic political discourse on poverty.

Although some studies have examined poverty discourse in China from a linguistic perspective (Tang and Mu, 2024; Chen, 2026), few have focused specifically on the metaphorical framing of poverty. Given the prominence of poverty discourse on China’s political agenda, one would expect extensive scholarly analysis of its metaphorical underpinnings. However, such studies are still lacking. Examining conceptual metaphors for poverty in Chinese political discourse can offer a valuable opportunity to deepen our understanding of not only how poverty is discursively constructed in China but also how social issues are conceptualized across different cultural and political contexts.

## 3 Methodology

### 3.1 Data collection

This study conducted a retrospective investigation into the major metaphors employed in the discourse of China’s poverty alleviation campaign. In November 2015, the government issued *the Decision of the Central Committee of the Communist Party of China and the State Council on Winning the Battle Against Poverty*, which marked the start of the campaign and reaffirmed the commitment of the Chinese government to eliminating poverty for all rural residents living below the current poverty line by 2020. In February 2021, the Chinese government declared the campaign a “complete victory,” announcing that nearly 100 million rural poor had been lifted out of poverty. The study focused on this specific time period and compiled a corpus comprising five key Chinese governmental reports issued at critical junctures during China’s poverty alleviation campaign, published between November 2015 and April 2021. The corpus comprises a total of 74,295 Chinese characters, which were segmented into 33,104 word tokens using *Jieba*, a Chinese word segmentation toolkit. Table 1 presents the composition of the corpus.

**Table 1:** Corpus of political discourse on China’s poverty alleviation.

Governmental report (GR)	Speech	Date of release	Chinese characters	Word count
GR1	*Decision of the Central Committee of the Communist Party of China and the State Council on Winning the Battle Against Poverty*	29 November 2015	13,228	5,718
GR2	*Guidelines of the CPC Central Committee and the State Council on the Three-Year Action Plan to Win the Battle Against Poverty*	15 June 2018	17,010	7,530
GR3	*Government Work Report Delivered at the Third Session of the 13th National People’s Congress*	22 May 2020	11,368	4,990
GR4	*Speech at the National Conference Summarizing Poverty Alleviation and Honoring Outstanding Contributors*	25 February 2021	8,869	4,078
GR5	The White Paper: *Poverty Alleviation: China’s Experience and Contribution*	6 April 2021	23,820	10,788
		Total	74,295	33,104

### 3.2 Data coding

Identification methods provide analysts with guidelines in metaphor identification. When identifying metaphors, metaphor analysts will not code based on their intuitions unless they are provided with methods and coding procedures.

A group of scholars has sought to develop a method for identifying metaphorically used words in discourse, that is, the “Method for Identifying Metaphorically Used Words in Discourse (MIP)” (Pragglejaz Group, 2007). MIP provides a reliable research method for determining whether words in contexts convey metaphorical meaning. It instructs analysts to identify the correspondence between the basic contemporary meaning and contextual meaning in discourses with the help of corpus-based dictionaries, thus identifying metaphorical units. Steen et al. (2010) put forward a new method called MIPVU in verbal metaphor identification and regarded it as a refinement and extension of MIP.

In this study, metaphor identification was conducted manually by the authors through a word-by-word reading of the corpus, following the MIPVU procedure. We determined whether a word was metaphorical or literal based on whether cross-domain mappings existed between the basic meaning and the contextual meaning of the word (Steen et al., 2010). We now demonstrate our metaphor identification procedure by applying MIPVU to the first sentence containing metaphorical expressions in GR1.

Example:

中共中央 国务院 关于 打赢 脱贫 攻坚战 的 决定 (*Decision of the CPC Central Committee and the State Council on Winning the Battle Against Poverty*) (From GR1).

We followed a set of guidelines (Steen et al., 2010, p. 25):

1) Find metaphor-related words (MRWs) by examining the text on a word-by-word basis.
2) When a word is used indirectly and that use may potentially be explained by some form of cross-domain mapping from a more basic meaning of that word, mark the word as metaphorically used (MRW).

In this example, we found two MRWs, namely “打赢 daying (to win)” and “攻坚战 gongjianzhan (the battle against…).” For “打赢 daying (to win),” the basic meaning is “to defeat the enemy on the battlefield or in combat,” while its contextual meaning is “to successfully achieve the goal of poverty alleviation.” Since there is a cross-domain mapping from the WAR domain to the POVERTY ALLEVIATION domain, it is identified as an MRW. For “攻坚战 gongjianzhan (the battle against…),” the basic meaning is “a military operation to attack enemy fortifications,” while its contextual meaning is “to resolve the most difficult tasks in poverty alleviation.” Again, the presence of cross-domain mapping from the WAR domain to the POVERTY ALLEVIATION domain warrants its identification as an MRW. We excluded other words in this sentence as MRWs because they do not involve cross-domain mappings between their basic and contextual meanings.

Based on the two MRWs identified in this sentence, we established conceptual mappings between the target domain of POVERTY ALLEVIATION and the source domain of WAR, thereby leading to the identification of the metaphor POVERTY ALLEVIATION IS WAR. The frequency of the WAR metaphor in this sentence is 2. When a sentence contains two or more metaphorical expressions, whether of the same or different types, we count each of them separately and classify them into their respective categories.

### 3.3 Inter-coder reliability

Although MIPVU improves the accuracy of research, the reliability of coding needs to be assessed in large corpora. Inter-coder reliability was assessed among two linguistically trained scholars who were instructed in conceptual metaphor theory and MIPVU. We worked independently to identify metaphors in the first governmental report and then discussed and resolved ambiguous cases.

The inter-coder reliability of the coding of the top four source domains was checked. The four source domains are WAR, PATH, COMPETITION, and HAT. Two coders’ coding results of frequencies of the top four source domains in the first government report were collected for the inter-coder reliability check. We considered 100% agreement if both coders agreed on the frequency of the specific source domain. The inter-coder reliability of each source domain was calculated in SPSS by statistically checking the κ value (Landis and Koch, 1977). All statistical analyses in this study were completed in IBM SPSS Statistics (version 26.0).

Results showed that reliability was “almost perfect” for coding the top four source domains, i.e., WAR (Cohen’s κ = 0.893), PATH (Cohen’s κ = 0.912), COMPETITION (Cohen’s κ = 0.882), and HAT (Cohen’s κ = 0.903).

## 4 Results

To address the first research question, we identified the source domains mapped to the target domains of POVERTY and POVERTY ALLEVIATION in the corpus. In this section, we report the frequency of the four most salient source domains and categorize the major metaphors based on the coding results.

### 4.1 Major source domains

In this sub-section, we report the four most salient source domains that mapped onto the target domains of POVERTY and POVERTY ALLEVIATION. The results show that the WAR metaphor is pervasively used, as the Chinese government has adopted the “War on Poverty” as a slogan and frequently invoked it in official discourse. This is followed by the PATH and COMPETITION metaphors in terms of their frequency. In addition, this study identified a novel metaphor, the HAT metaphor. Table 2 presents the frequency statistics for the top four source domains.

**Table 2:** Major source domains.

Source domain	Frequency	Proportion
WAR	402	75%
PATH	72	13%
COMPETITION	43	8%
HAT	19	4%

### 4.2 Major metaphors

The study categorizes the most salient metaphors in the corpus:

WAR metaphor: POVERTY ALLEVIATION IS A WAR;
PATH metaphor: POVERTY ALLEVIATION IS A PATH;
COMPETITION metaphor: POVERTY ALLEVIATION IS A COMPETITION;
HAT metaphor: POVERTY IS A HAT.

## 5 Findings and discussion

Drawing on the CMA framework, we conducted metaphor interpretation to examine how the Chinese government strategically employed specific metaphors, and metaphor explanation to elucidate why these dominant metaphors were employed in Chinese political discourse.

### 5.1 Metaphor interpretation

#### 5.1.1 WAR metaphor

The most salient metaphor identified in the dataset is the WAR metaphor, which is highly pervasive in Chinese political discourse, including in areas such as health communication (Gui, 2023; Tang and Sun, 2023) and economic policy (Sun et al., 2023; Zeng and Ahren, 2023). The Chinese government has frequently used the “War on Poverty” as an official slogan in its political discourse. The WAR metaphor allows us to understand a complex social governance initiative through the familiar experience of warfare. Within this war frame, Chinese civil servants are positioned as soldiers on the front lines in impoverished rural areas. At the same time, the government encourages every Chinese citizen to assume the role of a hero in this battle, striving for their own path out of poverty and toward a better life. These discursive roles are structured through a set of conceptual mappings and entailments, which we summarized in Table 3.

**Table 3:** Domain mappings within the WAR metaphor.

Source domain		Target domain
WAR	→	Poverty Alleviation
Battlefield	→	Rural Areas/Poverty-Stricken Counties
Soldier	→	Civil Servant/Social Worker
Hero	→	Chinese Citizen

Governmental reports provide ample evidence of the WAR metaphor, as the following examples demonstrate:

1. 增强 打赢 脱贫 攻坚战 的 使命感 紧迫感 (*Enhance the sense of mission and urgency in fighting and winning the war on poverty*) (From GR1)^1^.
2. 8 年来 ， 党中央 把 脱贫 攻坚 摆在 治国 理政 的 突出位置…组织 开展 了 声势浩大 的 脱贫 攻坚 人民战争 。*(Over the past eight years, the Central Committee of the CPC has placed poverty alleviation at the forefront of national governance… and has mobilized a large-scale people’s war on poverty)* (From GR4).
3. 动员 全 社会 科技 力量 投入 脱贫 攻坚 主战场， 开展 科技 精准 帮扶 行动 (The government mobilized the nation’s scientific and technological forces to join the main battlefield in poverty alleviation and carry out targeted science-based poverty reduction initiatives) (From GR2).
4. 在 脱贫 攻坚 没有 硝烟 的 战场 上， 广大党员 、 干部 以 热血 赴 使命 、 以 行动 践 诺言， 用 自己 的 辛劳 换来 贫困 群众 的 幸福 (On the smokeless battlefield of poverty alleviation, Party members and officials carried out their mission with devotion, and their actions honored their promises, bringing well-being to the poor through their own hard work) (From GR5).
5. 人民 是 真正 的 英雄 ， 激励 人民 群众 自力更生 、 艰苦奋斗 的 内生 动力， 对 人民 群众 创造 自己 的 美好生活 至关重要. (The people are the true heroes. Fostering their internal motivation for self-reliance and hard work is essential for them to create a better life for themselves) (From GR4).

Within the framework of conceptual metaphor theory, POVERTY ALLEVIATION IS A WAR [see Examples (1–2)] can be understood as a systematic cognitive mapping from the source domain of WAR onto the target domain of the social issue of POVERTY ALLEVIATION. The process of poverty alleviation is framed as a military campaign, as previous studies have found that the WAR metaphor serves to evoke urgency and mobilize collective action (Flusberg et al., 2018; Semino, 2020). Through this metaphor, the Chinese government frames poverty alleviation as a large-scale and tough, challenging campaign demanding strategic planning, heroic dedication, a pressing sense of urgency, and the confidence to overcome challenges.

The conceptual metaphors RURAL AREAS ARE BATTLEFIELDS and CIVIL SERVANTS ARE SOLDIERS [see Examples (3–4)] portray how Party members and officials in poverty-stricken counties working in impoverished counties are fighting at their posts in the “War on Poverty.” During this poverty alleviation campaign, the government mobilized millions of government officials to be stationed in poverty-stricken counties (Cai and Tang, 2023; Ruan et al., 2024). Their contributions were formally recognized at the commendation ceremony in 2021. These metaphors reflect the Chinese government’s commitment to the poverty alleviation initiative and its gratitude toward Party members and officials working on the front line.

Lakoff (1991) observed that U.S. politicians justified their intentions to go to war by framing “America as a hero” (Lakoff, 1991: 32) and by claiming that U.S. actions served the interests of the victims. By contrast, in China’s poverty alleviation discourse, the Chinese people are framed as heroes. This CHINESE CITIZENS ARE HEROES framing (see Example 5) not only effectively mobilizes Party members and officials at all levels but also makes the severity and significance of the campaign more comprehensible to ordinary citizens. We classify the HERO metaphor under the war frame, as “hero” consistently co-occurs with war-related terms in our corpus. In this context, the HERO metaphor helps convey the idea that the Chinese people themselves are the principal agents in the “War on Poverty.” This metaphor encourages individuals to take proactive steps toward self-reliance, embrace the spirit of hard work and diligent striving, and reduce excessive dependence on government support, thus fostering a sense of ownership and collective responsibility in national development efforts.

#### 5.1.2 PATH metaphor

The PATH metaphor is also salient in the discourse of poverty alleviation in the Chinese context. The PATH metaphor in poverty alleviation discourse can be understood as a systematic cognitive mapping process in which elements from the source domain of the social process of POVERTY ALLEVIATION are projected to the target domain of PATH. It uses the simple idea of moving along a path to help people understand this long and complex process. Scholars believe that the PATH metaphor sets a direction and provides a road map for short-term and long-term plans. It also creates a sense of progress and encourages collective efforts to overcome challenges (Zhang and Forceville, 2020). The PATH metaphor can be seen in the following governmental report:

1. 我们 立足 我国 国情 ， 把握 减贫 规律 ， 出台 一系列 超常规 政策 举措 ， 构建 了 一整套 行之有效 的 政策 体系 、 工作 体系 、 制度 体系 ， 走出 了 一条 中国 特色 减贫 道路 ， 形成 了 中国 特色 反 贫困 理论 。*(We have established a complete set of effective policy, operational, and institutional systems, forging a path to poverty alleviation with Chinese characteristics and developing a theory of poverty alleviation that reflects the Chinese context)* (From GR4).
2. 为 人类 减贫 探索 新 的 路径*(To explore a new path for poverty alleviation for humanity)* (From GR5)

In the poem *The Road Less Traveled* by Robert Frost, we understand the “road” as a choice. The road/path chosen by the Chinese government is officially referred to as the path to poverty alleviation with Chinese characteristics [see Example (1)]. This path is grounded in the theoretical system of Socialism with Chinese characteristics, which denotes the developmental and governance model adopted by China under the leadership of the CPC. This model integrates the basic tenets of Socialism with China’s specific historical, cultural, and material conditions. The poverty alleviation achievements by 2021 confirm that the Chinese path to poverty alleviation is the path to success.

The metaphor of POVERTY ALLEVIATION IS A PATH helps the Chinese government present its poverty alleviation policies as a clear and structured path to prosperity. Our analysis reveals a link between the PATH metaphor and the underlying SOURCE-PATH-GOAL schema. According to Johnson (1987), this image schema emerges from recurrent bodily experiences of movement and serves as a fundamental structure for reasoning about non-physical domains. When the government uses “path” to frame the process of poverty alleviation, it reflects a cognitive mapping of this basic image schema onto the abstract domain of social development. The mapping of the SOURCE-PATH-GOAL schema onto poverty alleviation enables the government to cast an otherwise complex policy process as a coherent, goal-oriented trajectory, which renders the abstract notion of development as a more intuitive and communicable narrative.

In the *White Paper Poverty Alleviation: China’s Experience and Contribution* (GR5), the Chinese government also stated that this new path for poverty alleviation is for all humanity [see Example (2)]. This further corroborates China’s resolve to build a community with a shared future for mankind.

#### 5.1.3 COMPETITION metaphor

The COMPETITION metaphor is identified from the corpus. It is a conceptual metaphor that uses sports competitions or competitive contests as the source domain. Previous studies have examined the COMPETITION metaphor in marketing and business contexts, showing that companies use it to emphasize the competitive nature of the business environment (Koller, 2004; Downing et al., 2013). By mapping structures such as the rules, objectives, and participant relationships from competitions onto social, economic, technological, and other fields, it explains and promotes ongoing development in specific domains. The following are some examples of the COMPETITION metaphor:

1. 我国 扶贫开发 已 进入 啃 硬骨头 、 攻坚 拔寨 的 冲刺 期 *(China’s poverty alleviation drive has entered the final sprint phase, a period that demands tackling the toughest challenges to secure ultimate victory)* (From GR1).
2. 加强 教育 扶贫， 不让 孩子 输在 起跑线 上*(We strengthened education-based poverty alleviation to ensure that no child would be left behind at the starting line)* (From GR5).

The metaphor POVERTY ALLEVIATION IS A COMPETITION in both examples frames the process of poverty alleviation as a competitive endeavor (Shields and Bredemeier, 2011), mapping the source domain of COMPETITION onto the target domain of POVERTY ALLEVIATION. As Goatly (2007, p. 336) notes, “humans are involved in a competition for survival of themselves and their progeny.” This competitive view of human nature has led to the widespread use of SPORTS/COMPETITION/RACE metaphors across various discursive contexts (Ganesh and Zorn, 2011; Sun and Jiang, 2014; Jawad and Noori, 2021). The COMPETITION metaphor drives time pressure by leveraging the sense of time constraints and the awareness of an endpoint inherent in competitions. It reinforces the urgency of policy implementation and promotes the timely accomplishment of tasks.

#### 5.1.4 HAT metaphor

The HAT metaphor is also seen in China’s poverty alleviation discourse. The keyword “脱贫摘帽 tuopin zhaimao” evokes a cognitive mapping in which the “hat” serves as the source domain. By linking the “hat” to social identity, this metaphor helps people understand how poverty status can be shed and who is responsible for its removal. The HAT metaphor can be manifested in the following government report:

1. 脱贫 摘帽 不是 终点， 而是 新 生活 、 新 奋斗 的 起点 (Poverty alleviation is not the end, but the starting point of a new life and a new struggle) (From GR4).
2. …明确 到 2020 年 现行标准 下 农村 贫困人口 实现 脱贫 、 贫困县 全部 摘帽 、 解决 区域性 整体 贫困 的 目标 任务 (…clearly defining the objectives and tasks of lifting all rural residents living below the current poverty line out of poverty by 2020, removing all poverty-stricken counties from the poverty list, and eliminating regional overall poverty) (From GR5).

In Chinese culture, the hat carries various symbolic meanings. In ancient China, “hat” served as a symbol of social status, primarily used by the upper class, with its symbolic meaning far exceeding its practical function as headwear. Over the course of different historical periods in China, the symbolic meaning of “hat” varied. In modern China, the word “hat” (帽子 màozi) carries both positive and negative connotations in the Chinese language. For example, “戴高帽 dai gaomao” is a Chinese idiom that literally means “wearing a very tall hat.” Figuratively, it refers to speaking flattering or complimentary words to others or offering excessive praise to curry favor (Wu, 2016).

The Chinese government strategically employs the metaphor “POVERTY IS A HAT” to transform poverty from an abstract concept into a clear and manageable symbol. The Chinese term “hat” (帽子màozi) signifies social rank or official status while also carrying a negative connotation. The metaphorical labeling of a county as “poverty-stricken” (i.e., “wearing the poverty hat”) prompts its residents to work toward removing this designation. This framing not only makes policies easier to communicate but also helps people focus more on their goals. However, the HAT metaphor is rarely used in Western poverty discourse.

### 5.2 Metaphor explanation

The analysis in the previous section shows how metaphors were used in China’s poverty alleviation discourse. This study also provides explanations for the intensive use of major metaphors to answer the second research question.

First, metaphors are considered to have played a significant discursive role in facilitating China’s poverty alleviation efforts. As noted by Charteris-Black (2021, p. 51), the WAR metaphor serves to “heighten the awareness of the danger,” thereby capturing public attention and motivating collective action (Flusberg et al., 2018). Flusberg et al. (2018) also observed that the declaration of a “War on Poverty” successfully drew public attention to the issue and facilitated the introduction of major anti-poverty policies and legislation. This study also suggests that the WAR metaphor in Chinese political discourse on poverty alleviation constructs poverty as an urgent threat, captures public attention, and prompts action on critical social issues. Within China’s poverty alleviation discourse, the use of the WAR metaphor conveys a sense of gravitas, ensuring that the seriousness of the situation is recognized. Within this war frame, every individual is positioned as a hero, motivating individuals to pursue self-reliance, embody the ethos of hard work and perseverance, and reduce reliance on governmental assistance. The finding regarding the pervasive use of the COMPETITION metaphor in Chinese political discourse also aligns with earlier research (Koller, 2004; Downing et al., 2013; Musolff, 2022). As these studies indicate, the COMPETITION metaphor generates time pressure, heightens policy urgency, and facilitates timely achievement of objectives. In contrast to earlier research, this study uncovers a culturally salient HAT metaphor. Because “hat” carries negative connotations in Chinese, the HAT metaphor elicits a positive response from the public. Wearing the “hat” signifies belonging to a disadvantaged group, while removing it represents victory in the “War on Poverty.” Like the WAR and COMPETITION metaphors, the HAT metaphor likewise facilitates China’s poverty alleviation success.

Second, metaphors help the Chinese government build a positive national image and present itself as a model in international poverty alleviation. The use of the PATH metaphor in political discourse is not confined to Western settings (Cibulskiene, 2012; Hampl, 2022); it has also been observed in Chinese political communication (Zhang and Forceville, 2020; Chang, 2023). In the Chinese context, the PATH metaphor frames poverty alleviation as a collective journey toward shared prosperity. This framing not only facilitates policy communication domestically but also shapes China’s international identity as a responsible contributor to global poverty reduction. Phrases like “working together to build a global community with a shared future free from poverty” in Chinese governmental reports are often paired with calls to “forge a global path toward poverty reduction,” elevating the PATH metaphor into a global initiative. The global relevance of this framing is reinforced by the recognition of China’s poverty alleviation efforts by the international community. Notably, United Nations Secretary-General António Guterres lauded China’s achievement as “the greatest anti-poverty achievement in history” (United Nations, 2019). The WAR metaphor frames poverty alleviation as a major campaign with clear objectives, strong leadership, and a resolute determination to succeed (Flusberg et al., 2018). It thus projects an image of a government that honors its commitments and is capable of mobilizing national resources to address complex challenges. With the success of the “War on Poverty,” the Chinese government demonstrates to the world that it serves as a capable leader on the path to prosperity, guiding its people toward freedom from poverty.

## 6 Conclusion

Metaphors have consistently shaped the discourse on China’s fight against poverty. This study analyzes the metaphorical framing of the poverty alleviation campaign, examining how and why major metaphors are used in the Chinese context. Drawing on five governmental reports, the analysis identifies WAR, PATH, COMPETITION, and HAT metaphors following the MIPVU procedure and discusses their discursive functions and strategic motivations. This study maintains that metaphors have played a significant discursive role in facilitating China’s poverty alleviation efforts and simultaneously projecting a positive national image and positioning the country as a model in international poverty reduction.

This study offers an in-depth analysis of the mechanisms and social effects of political metaphors. It contributes to metaphor studies in the Chinese context while also providing insights for policy practice and international dialogue. Although this study identified similar patterns of metaphor use, particularly the WAR, COMPETITION, and PATH metaphors in poverty alleviation discourse, it also uncovered a culturally specific metaphorical frame, the HAT metaphor, that is rarely employed in Western contexts. This further confirms that metaphor use and interpretation are context-based (Kövecses, 2015). The findings are believed to offer practical insights for professionals in political communication, policy formulation, and international development.

## 7 Limitations and future directions

Despite its contributions, this study is subject to several limitations that should be acknowledged. The most significant limitation pertains to the size and scope of the corpus used in the analysis. Although the dataset was carefully constructed to ensure quality and relevance for the research questions, its relatively modest scale inevitably constrains the generalizability and robustness of our findings. A larger corpus would have enabled a more robust analysis, revealing subtler patterns and supporting stronger conclusions. Consequently, the findings of this study should be interpreted as indicative of trends within the specific dataset examined, rather than as universally applicable truths.

This limitation also paves the way for future research. A valuable next step would be to replicate this analysis with a larger, more diverse dataset. This would help verify the robustness of our findings. A larger corpus would also allow for more sophisticated statistical and machine learning methods, which can uncover complex patterns beyond the scope of a smaller study.

## Data Availability

The raw data supporting the conclusions of this article will be made available by the authors, without undue reservation.

## Footnotes

1 In the examples throughout the manuscript, boldface is used to highlight metaphor-related words (MRWs) that instantiate the specific metaphor under discussion. Examples may contain additional metaphorical expressions that are not highlighted, as they fall outside the focus of the analysis.

## References

[ref1] Bates B. R. (2020). The (in) appropriateness of the WAR metaphor in response to SARS-CoV-2: a rapid analysis of Donald J. Trump’s rhetoric. Front. Commun. 5:50. doi: 10.3389/fcomm.2020.00050

[ref2] Cai C. Tang N. (2023). China’s campaign-style implementation regime: how is “targeted poverty alleviation” being achieved locally? J. Chin. Polit. Sci. 28, 645–669. doi: 10.1007/s11366-022-09823-1

[ref3] Chang J. (2023). Cognitive study of path metaphor and military metaphor in political discourse. Critique Semiotics 1, 85–104.

[ref4] Charteris-Black J. (2004). Corpus Approaches to Critical Metaphor Analysis. Basingstoke: Palgrave Macmillan.

[ref5] Charteris-Black J. (2006). Britain as a container: immigration metaphors in the 2005 election campaign. Discourse Soc. 17, 563–581. doi: 10.1177/0957926506066345

[ref6] Charteris-Black J. (2011). Politicians and Rhetoric: The Persuasive power of Metaphor. 2nd Edn. Basingstoke: Palgrave Macmillan.

[ref7] Charteris-Black J. (2018). Analysing Political Speeches: Rhetoric, Discourse and Metaphor. London: Macmillan International Higher Education.

[ref8] Charteris-Black J. (2019). Metaphors of Brexit: No Cherries on the Cake. Cham, Switzerland: Springer Nature.

[ref9] Charteris-Black J. (2021). Metaphors of Coronavirus: Invisible enemy or zombie Apocalypse? Basingstoke: Palgrave Macmillan.

[ref10] Chen L. (2026). Sketching poverty: a corpus-based analysis of media representation of poverty in China in the post-poverty-alleviation era. Corpus Pragmat. 10:25. doi: 10.1007/s41701-025-00227-z

[ref11] Chilton P. (2025). Analysing Political Discourse: Theory and Practice. 2nd Edn. New York, NY: Routledge.

[ref12] Cibulskiene J. (2012). The development of the journey metaphor in political discourse: Time-specific changes. Metaphor Soc. World 2, 131–153. doi: 10.1075/msw.2.2.01cib

[ref13] David O. Matlock T. (2018). Cross-linguistic automated detection of metaphors for poverty and cancer. Lang. Cogn. 10, 467–493. doi: 10.1017/langcog.2018.11

[ref14] Dodge E. K. (2016). A deep semantic corpus-based approach to metaphor analysis: a case study of metaphorical conceptualizations of poverty. Constr. Frames 8, 256–294.

[ref15] Downing H. Kraljevic B. Mujic B. Nύnez-Perucha B. (2013). Metaphorical creativity and recontextualization in multimodal advertisements on e-business across time. Metaphor Soc. World 3, 199–219. doi: 10.1075/msw.3.2.05hid

[ref16] Fairclough N. (1995). Critical Discourse Analysis: The Critical Study of Language. London: Pearson Education Limited.

[ref17] Ferrari F. (2007). Metaphor at work in the analysis of political discourse: investigating a “preventive war” persuasion strategy. Discourse Soc. 18, 603–625. doi: 10.1177/0957926507079737

[ref18] Flusberg S. J. Matlock T. Thibodeau P. H. (2018). War metaphors in public discourse. Metaphor. Symb. 33, 1–18. doi: 10.1080/10926488.2018.1407992

[ref19] Fu F. (2024). Examining Chinese people’s protective health behavior in the context of COVID-19 metaphors. Curr. Psychol. 43, 33720–33728. doi: 10.1007/s12144-024-06369-8

[ref20] Ganesh S. Zorn T. (2011). Running the race: competition discourse and broadband growth in Aotearoa New Zealand. Media Cult. Soc. 33, 725–742. doi: 10.1177/0163443711404465

[ref21] Goatly A. (2007). Washing the brain: Metaphor and hidden Ideology (Vol. 23). Amsterdam: John Benjamins Publishing.

[ref22] Gui L. (2023). Media framing and public perception: the use of war metaphor in times of COVID-19. J. Multicult. Discourses 18, 140–153. doi: 10.1080/17447143.2023.2292543

[ref23] Hampl M. (2022). The use of the PATH and FORCE image schemas in Barack Obama's counterterrorism discourse against ISIL. Crit. Discourse Stud. 19, 274–289. doi: 10.1080/17405904.2020.1855220

[ref24] Jawad H. Noori A. (2021). A cognitive-semantic study of conceptual metaphors in English news reports. J. Univ. Human Dev. 7:12. doi: 10.21928/juhd.v7n1y2021.pp12-34

[ref25] Johnson M. (1987). The body in the mind: The Bodily basis of Meaning, Imagination, and Reason. Chicago: University of Chicago Press.

[ref26] Koller V. (2004). Metaphor and Gender in Business media Discourse: A Critical Cognitive Study. Basingstoke: Palgrave Macmillan.

[ref28] Kövecses Z. (2015). Where Metaphors come from: Reconsidering context in Metaphor. Oxford: Oxford University Press.

[ref29] Lakoff G. (1991). Metaphor and war: the metaphor system used to justify war in the Gulf. Peace Res. 23, 25–32.

[ref30] Lakoff G. (1996). Moral Politics: How Liberals and Conservatives Think. Chicago: University of Chicago Press.

[ref32] Lakoff G. Johnson M. (1980). Metaphors we live by. Chicago: Chicago University Press.

[ref33] Landis J. R. Koch G. G. (1977). The measurement of observer agreement for categorical data. Biometrics 33, 159–174. doi: 10.2307/2529310 843571

[ref34] Liu M. Feng X. Wang S. Qiu H. (2020). China’s poverty alleviation over the last 40 years: successes and challenges. Aust. J. Agric. Resour. Econ. 64, 209–228. doi: 10.1111/1467-8489.12353

[ref35] Lu W. L. Ahrens K. (2008). Ideological influence on building metaphors in Taiwanese presidential speeches. Discourse Soc. 19, 383–408. doi: 10.1177/0957926508088966

[ref36] Musolff A. (2006). Metaphor scenarios in public discourse. Metaphor Symbol 21, 23–38. doi: 10.1207/s15327868ms2101_2

[ref37] Musolff A. (2012). The study of metaphor as part of critical discourse analysis. Crit. Discourse Stud. 9, 301–310. doi: 10.1080/17405904.2012.688300

[ref38] Musolff A. (2016). Political Metaphor Analysis: Discourse and Scenarios. London: Bloomsbury Publishing.

[ref39] Musolff A. (2022). “‘War against COVID-19’: is the pandemic management as war metaphor helpful or hurtful?” in Pandemic and Crisis Discourse: Communicating COVID-19 and public Health Strategy, eds. Musolff A. Breeze R. Kondo K. Vilar-Lluch S. (London: Bloomsbury Publishing), 307–320.

[ref40] Pragglejaz Group (2007). MIP: a method for identifying metaphorically used words in discourse. Metaphor Symbol 22, 1–39. doi: 10.1080/10926480709336752

[ref41] Ruan R. Liu L. Zheng F. Liu S. (2024). How to channel state resources into rural society under the withering of grassroots governance: lessons from the targeted poverty alleviation “pairing-up” support system in China. J. Rural. Stud. 109:103331. doi: 10.1016/j.jrurstud.2024.103331

[ref42] Semino E. (2020). “Not soldiers but fire-fighters”–metaphors and COVID-19. Health Commun. 36, 50–58. doi: 10.1080/10410236.2020.1844989, 33167731

[ref43] Shields D. Bredemeier B. (2011). Contest, competition, and metaphor. J. Philos. Sport 38, 27–38. doi: 10.1080/00948705.2011.9714547

[ref44] State Council Information Office of the People’s Republic of China (2021). Poverty Alleviation: China’s Experience and Contribution. Available online at: https://en.qstheory.cn/2021-04/07/c_676783.htm

[ref45] Steen G. J. Dorst A. G. Herrmann J. B. Kaal A. A. Krennmayr T. Pasma T. (2010). A Method for linguistic Metaphor Identification: From MIP to MIPVU. Amsterdam: John Benjamins.

[ref46] Sun Y. Jiang J. (2014). Metaphor use in Chinese and US corporate mission statements: a cognitive sociolinguistic analysis. Engl. Spec. Purp. 33, 4–14. doi: 10.1016/j.esp.2013.07.005

[ref47] Sun Y. Kong D. Zhou C. (2023). Economy or ecology: metaphor use over time in China’s government work reports. Lang. Cogn. 15, 551–573. doi: 10.1017/langcog.2023.18

[ref48] Tang Q. Mu Y. (2024). Corpus-based diachronic analysis on the representations of China’s poverty alleviation in people’s daily. Corpus-Based Studies Across Humanities 1, 245–266. doi: 10.1515/csh-2023-0019

[ref49] Tang P. Sun i. (2023). From a war of defense to conventional wars: military metaphors for COVID-19 containment in Chinese documentaries. Rev. Cogn. Linguist. 21, 444–468. doi: 10.1075/rcl.00141.tan

[ref50] Thompson S. (2018). “Politics without metaphors is like a fish without water,” in Metaphor: Implications and Applications, eds. Mio J. S. Katz A. N. (Mahwah, New Jersey: Psychology Press), 185–201.

[ref51] United Nations (2019). Helping 800 million People escape Poverty was Greatest such Effort in History, Says Secretary-General, on Seventieth Anniversary of China’s Founding (Press Release SG/SM/19779). Available online at: https://press.un.org/en/2019/sgsm19779.doc.htm

[ref52] Wu M. (2016). The structure “X Mao (zi)” and its cultural connotation. Chinese Culture and Teaching in the World 0, 99–108.

[ref53] Zeng W. Ahren K. (2023). Corpus-based metaphorical framing analysis: WAR metaphors in Hong Kong public discourse. Metaphor. Symb. 38, 254–274. doi: 10.1080/10926488.2022.2158088

[ref54] Zeng W. Burgers C. Ahrens K. (2021). Framing metaphor use over time: “free economy” metaphors in Hong Kong political discourse (1997-2017). Lingua 252:102955. doi: 10.1016/j.lingua.2020.102955

[ref55] Zhang C. Forceville C. (2020). Metaphor and metonymy in Chinese and American political cartoons (2018-2019) about the Sino-US trade conflict. Pragmatics Cogn. 27, 474–499. doi: 10.1075/pc.20013.zha

